# Circulating Reelin promotes inflammation and modulates disease activity in acute and long COVID-19 cases

**DOI:** 10.3389/fimmu.2023.1185748

**Published:** 2023-06-27

**Authors:** Laurent Calvier, Aleksandra Drelich, Jason Hsu, Chien-Te Tseng, Yair Mina, Avindra Nath, Maria Z. Kounnas, Joachim Herz

**Affiliations:** ^1^Department of Molecular Genetics, University of Texas (UT) Southwestern Medical Center, Dallas, TX, United States; ^2^Center for Translational Neurodegeneration Research, UT Southwestern Medical Center, Dallas, TX, United States; ^3^Department of Biochemistry, Cellular, and Molecular Biology, University of Texas Medical Branch (UTMB) Health, Galveston, TX, United States; ^4^National Institute of Neurological Disorders and Stroke, National Institutes of Health, Bethesda, MD, United States; ^5^Sackler Faculty of Medicine, Tel-Aviv University, Tel-Aviv, Israel; ^6^Reelin Therapeutics Inc., San Diego, CA, United States; ^7^Department of Neuroscience, UT Southwestern Medical Center, Dallas, TX, United States; ^8^Department of Neurology and Neurotherapeutics, UT Southwestern Medical Center, Dallas, TX, United States

**Keywords:** Reelin, endothelial dysfunction, adhesion markers, cytokine storm, inflammation, leukocyte, COVID-19, long COVID

## Abstract

Thromboembolic complications and excessive inflammation are frequent in severe COVID-19, potentially leading to long COVID. In non-COVID studies, we and others demonstrated that circulating Reelin promotes leukocyte infiltration and thrombosis. Thus, we hypothesized that Reelin participates in endothelial dysfunction and hyperinflammation during COVID-19. We showed that Reelin was increased in COVID-19 patients and correlated with the disease activity. In the severe COVID-19 group, we observed a hyperinflammatory state, as judged by increased concentration of cytokines (IL-1α, IL-4, IL-6, IL-10 and IL-17A), chemokines (IP-10 and MIP-1β), and adhesion markers (E-selectin and ICAM-1). Reelin level was correlated with IL-1α, IL-4, IP-10, MIP-1β, and ICAM-1, suggesting a specific role for Reelin in COVID-19 progression. Furthermore, Reelin and all of the inflammatory markers aforementioned returned to normal in a long COVID cohort, showing that the hyperinflammatory state was resolved. Finally, we tested Reelin inhibition with the anti-Reelin antibody CR-50 in hACE2 transgenic mice infected with SARS-CoV-2. CR-50 prophylactic treatment decreased mortality and disease severity in this model. These results demonstrate a direct proinflammatory function for Reelin in COVID-19 and identify it as a drug target. This work opens translational clinical applications in severe SARS-CoV-2 infection and beyond in auto-inflammatory diseases.

## Introduction

Substantial knowledge has been gained in a short period about COVID-19, due to this disease’s propagation and economic burden worldwide. Owing to intense research efforts, the major pathophysiological mechanisms are fairly well understood. The main causes of death from acute SARS-Cov-2 infection are adult respiratory distress syndrome (ARDS) and thromboembolic complications (1–3). ARDS is characterized by acute and diffuse inflammatory damage into the alveolar-capillary barrier associated with vascular dysfunction marked by increased permeability, reduced compliance, and intracapillary thrombosis (4). This adverse condition compromises gas exchange, causing hypoxemia, and damages organs due to a lack of oxygen. In the most severe cases, this infection and inflammatory environment progress to a hyperinflammatory state or a cytokine storm. It manifests itself by an overproduction of inflammatory molecules, such as interleukin (IL) -6 and -10, tumor necrosis factor α (TNF-α), or interferon γ (IFN-γ) for example (5–7).

Following initial SARS-Cov-2 infection, approximately 3/4 of the hospitalized patients and 1/3 of the out-of-hospital patients experience persistent symptoms for several weeks or months, regardless of their viral status (8). These persistent symptoms are known as post-acute sequelae of COVID-19 (PASC), or long- COVID, which is defined as a variety of new, returning, or ongoing health problems people can experience for four or more weeks following initial SARS-CoV-2 infection (Centers for Disease Control and Prevention, 2021)(9). Although most PASC patients no longer have a viral load, they suffer from various persistent disorders such as cardiovascular, respiratory, articular, neurologic, and psychiatric. Symptoms vary between individuals and include fatigue, hair loss, cough, shortness of breath, dyspnea, joint pain, loss of smell and taste, attention disorder, headache, memory loss, gastrointestinal distress, cerebrovascular disorders, dysrhythmias, pericarditis, myocarditis, heart failure, and thromboembolic disease (8–13). Long COVID risk increases with age and has been associated with acute disease severity (14), but the exact mechanism behind the prolonged symptoms still needs to be identified. One hypothesis posits that the transition from acute to long COVID may be due to endothelial injury and dysfunction, cytokine storm, dysregulation of the immune response, and the ability of coronavirus or fragments of the virus to evade the immune system (8–13).

We have previously identified a circulating protein, Reelin, that is central in the initiation and propagation of endothelial dysfunction with additional prothrombotic function (15, 16). These two mechanisms are key in acute and long COVID (4), suggesting a role for Reelin in this infection. In endothelial cells, adhesion and permeability to leukocytes are greatly regulated *via* the NF-κB pathway (17). NF-κB target genes include “rolling” molecules such as E-selectin and adhesion molecules such as intercellular adhesion molecule 1 (ICAM-1) or vascular cell adhesion protein 1 (VCAM-1), but also cytokines such as IL-1, -6, TNFα, and chemokines such as C-C motif chemokine ligand (CCL)-2, -8, C-X-C motif chemokine ligand (CXCL)-2, and C-X3-C motif chemokine ligand (CX3CL)-1. Endothelial adhesion followed by endothelial transmigration are primarily regulated by NF-κB target genes (17). However, NF-κB has major additional functions and direct obliteration of this pathway would result in severe side effects. We have identified a regulator, Reelin, that controls NF-κB activation and the expression of its target genes required for efficient endothelial-leukocyte adhesion and endothelial permeability. In atherosclerosis (18, 19) or multiple sclerosis (20, 21), Reelin promotes inflammatory cell recruitment and inflammation by increasing the expression of leukocyte-endothelial adhesion markers (E-selectin, ICAM-1, and VCAM-1) on endothelial cells. Expression of these inflammatory mediators is increased by Reelin *via* NF-κB signaling through its receptor, the apolipoprotein E receptor 2 (ApoER2 or LRP8) which is a member of the low-density lipoprotein receptor (LDLR) family (22). In addition to this proinflammatory function, some studies suggest that reelin may have a role in thrombosis by affecting platelet function and blood coagulation (23–26). It has been reported that Reelin can interact with platelets through different receptors (including ApoER2) and enhance platelet spreading and aggregation to form a blood clot. These studies support an additional function for plasma Reelin as a pro-thromboembolic factor. However, a role for Reelin in COVID-19 remains yet to be determined.

In this study, we hypothesized that Reelin promotes endothelial dysfunction and participates in the propagation of hyperinflammation during COVID-19. To this end, we have investigated the inflammatory response in acute and long COVID patients, correlated inflammatory markers with Reelin levels, and tested Reelin inhibition in SARS-CoV-2-infected hACE2 transgenic mice.

## Material and methods

### Study populations

Serum from the acute COVID cohort was obtained from the UT Southwestern SARS-CoV-2 Biorepository and the long COVID cohort from Dr. Avindra Nath. The study involves only secondary research using data or biospecimens not collected specially for this study and the specimens or data were provided without identifiable information. Thus, it is considered as “Not Human Research,” which does not require institutional review board approval. Detailed characteristics of each cohort can be found in **Tables 1** and **2**.

**Table 1:** Biomarker concentrations in serum from patients a with mild or severe form of Covid-19.

Biomarker	Control	Mild Covid	Severe Covid	P value
n (total)	15	15	15	
n (female)	7	7	6	
n (male)	8	8	9	
Age	54.7 ± 3.1	53.1 ± 4.2	59.1 ± 3.2	
Disease severity				
Hospitalization	0%	100%	100%	
Pneumonia	0%	0%	100%	
ARDS	0%	0%	100%	
ICU	0%	0%	100%	
Ventilator	0%	0%	100%	
ECMO	0%	0%	13.30%	
Biomarker (ng/ml)				
Reelin	10.4 ± 1.3	22.9 ± 3.9	48.4 ± 11.0	*CT vs MC; **CT vs SC
Adhesion markers (ng/ml)				
E-selectin	17.6 ± 4.0	17.6 ± 2.1	25.2 ± 2.6	*CT vs SC
P-selectin	77.9 ± 15.2	98.7 ± 17.2	214.5 ± 87.6	
ICAM-1	48.6 ± 15.3	37.8 ± 5.9	138.5 ± 29.6	**CT vs SC; **MC vs SC
Cytokines (pg/ml)				
IFN-α	3.2 ± 3.0	5.5 ± 3.4	8.9 ± 4.2	
IFN-γ	9.1 ± 2.1	12.6 ± 5.2	14.6 ± 2.6	
TNF-α	11.4 ± 1.1	11.6 ± 1.8	18.6 ± 2.6	
IL-1α	1.3 ± 0.6	0.8 ± 0.3	3.8 ± 1.5	*CT vs SC; *MC vs SC
IL-1β	7.2 ± 4.8	5.7 ± 4.3	9.1 ± 2.9	P=0.07 for CT vs SC
IL-4	11.4 ± 1.2	18.2 ± 4.1	24.8 ± 3.8	**CT vs SC
IL-6	< detection limit	< detection limit	530.2 ± 341.5	Non-applicable
IL-8	5.0 ± 2.4	0.5 ± 0.3	24.4 ± 18.9	
IL-10	< detection limit	< detection limit	< detection limit	
IL-12p70	251.8 ± 33.0	248.8 ± 25.1	299.6 ± 28.4	
IL-17A	5.1 ± 2.4	3.1 ± 0.8	11.4 ± 2.6	*CT vs SC; *MC vs SC
Chemokines (pg/ml)				
IP-10 (CXCL10)	12.2 ± 2.2	34.4 ± 11.0	70.9 ± 16.5	**CT vs SC; *MC vs SC
MCP-1 (CCL2)	39.0 ± 9.7	42.0 ± 10.5	90.9 ± 24.2	
MIP-1α (CCL3)	5.8 ± 4.2	0.9 ± 0.2	3.8 ± 1.3	
MIP-1β (CCL4)	70.5 ± 10.4	59.0 ± 6.6	124.2 ± 17.3	*CT vs SC; **MC vs SC

CT, control; MC, mild Covid; SC, severe Covid; ARDS, acute respiratory distress syndrome; ICU, intensive care unit; ECMO, extracorporeal membrane oxygenation; *p<0.05, **p<0.01.

**Table 2:** Biomarker concentrations in serum from patients with a long form of Covid-19.

Biomarker	Control	Long Covid	P value
n (total)	12	12	
n (female)	10	10	
n (male)	2	2	
Age	50.3 ± 2.6	45.4 ± 3.0	
Biomarker (ng/ml)			
Reelin	10.3 ± 1.6	9.8 ± 1.1	
Adhesion markers (ng/ml)			
E-selectin	17.0 ± 1.6	12.5 ± 2.4	
P-selectin	86.6 ± 18.0	93.3 ± 13.7	
ICAM-1	25.6 ± 5.3	20.2 ± 3.0	
Cytokines (pg/ml)			
IFN-α	< detection limit	< detection limit	
IFN-γ	6.2 ± 0.6	5.0 ± 0.8	
TNF-α	10.2 ± 1.0	10.9 ± 1.0	
IL-1α	1.3 ± 0.7	2.3 ± 0.9	
IL-1β	0.7 ± 0.3	0.3 ± 0.2	
IL-4	9.5 ± 0.7	10.1 ± 0.5	
IL-6	< detection limit	< detection limit	
IL-8	5.5 ± 3.0	14.3 ± 7.3	
IL-10	< detection limit	< detection limit	
IL-12p70	231.4 ± 22.4	195.1 ± 24.7	
IL-17A	2.7 ± 0.8	1.0 ± 0.3	P=0.06
Chemokines (pg/ml)			
IP-10	10.4 ± 1.0	8.4 ± 1.9	P=0.07
MCP-1	32.2 ± 11.3	12.8 ± 2.3	
MIP-1α	7.1 ± 5.2	0.8 ± 0.2	
MIP-1β	82.7 ± 10.2	93.4 ± 14.6	

### Biomarker assays

Reelin ELISA was performed on human serum samples diluted at 1:30 and run according to the manufacturer’s instructions (LSBio, N-Terminal part: LS-F7023). The inflammatory multiplexing panel was performed on human serum samples using the Inflammation 20-Plex Human ProcartaPlex™ Panel (Thermo Fisher Scientific, Catalog number: EPX200-12185-901) according to the manufacturer’s instructions. All protein markers for which more than 50% of the measurements fell into the detection range of the standard curves were considered valid.

### SARS-CoV-2 infection models

All experiments involving infectious viruses were carried out at the University of Texas Medical Branch (UTMB), Galveston, TX, under an animal use and care protocol approved by the UTMB IACUC in AALAC-accredited animal biosafety level 3 and biosafety level 3 laboratories. The effect of anti-Reelin treatment for inhibiting SARS-CoV-2 infection *in vivo* was determined using transgenic mice globally expressing human angiotensin-converting enzyme 2 (hACE2 Tg), AC70 line (Taconic Biosciences). Briefly, Tg mice were separated into two groups, the controls injected with unspecific mouse IgG 100µg/mouse and the treated injected with anti-Reelin mouse antibody (CR-50, prepared in our laboratory) 100µg/mouse. Injections were done *via* the intraperitoneal (i.p.) route 72 hours before and at day 0, 3 and 6 after challenge intranasally (i.n.) with ~5 TCID50 (~1.56 LD50) of SARS-CoV-2 (US_WA-1/2020 isolate) in 60µl of Eagle’s Minimum Essential Medium (EMEM) supplemented with 2% heated-inactivated fetal bovine serum (FBS) (M-2). Animals were monitored daily for body weight changes and clinical signs until they met the criteria for euthanasia using a scoring system as described before (27). To compare plasma Reelin expression, we also employed a hamster model. Briefly, hamsters (8-10-week old) obtained from Charles River (Houston, Texas), were challenged i.n. with ~10^5 TCID50 of SARS-CoV-2 in 100µl of M-2.

### Western blot

An equal volume of plasma (diluted 1/10 in PBS) was loaded into each lane of a 4-12% Tris gel (BioRad) and subjected to electrophoresis followed by transfer on nitrocellulose-membranes (BioRad). The membranes were stained with Ponceau S to ensure equal loading, blocked for 1h (milk powder 5% in TBS/tween 0.1- 0.2%), and incubated with primary antibodies (G10 anti-Reelin, made in-house). Secondary HRP-antibody binding was visualized by ECL or ECL plus chemiluminescent (Amersham). After densitometric analyses with ImageJ, optical density values were expressed as arbitrary units (28–30).

### Statistical analysis

The n values are specified in each legend. The GraphPad Prism software was used to run all the statistical analyses. Values from multiple experiments are expressed as means ± SEM. Normality was tested using the Kolmogorov-Smirnov test. Statistical significance was determined for multiple comparisons using one-way analysis of variance (ANOVA) followed by Tukey’s multiple comparisons (for normal distribution) or Kruskal-Wallis (for non-normal distribution) test. Student’s t-test (for normal distribution) or Mann-Whitney (for non-normal distribution) were used for comparisons of two groups. The correlations were calculated by linear regression (Pearson’s r). The survival curves were tested with log-rank (Mantel-Cox test). P < 0.05 was considered significant, with: * p < 0.05; ** p < 0.01; p < 0.001 and lower are not marked specifically and included in ** p < 0.01.

## Results

### Circulating Reelin is increased and correlates with disease severity in patients with acute COVID-19

**Figure 1:**
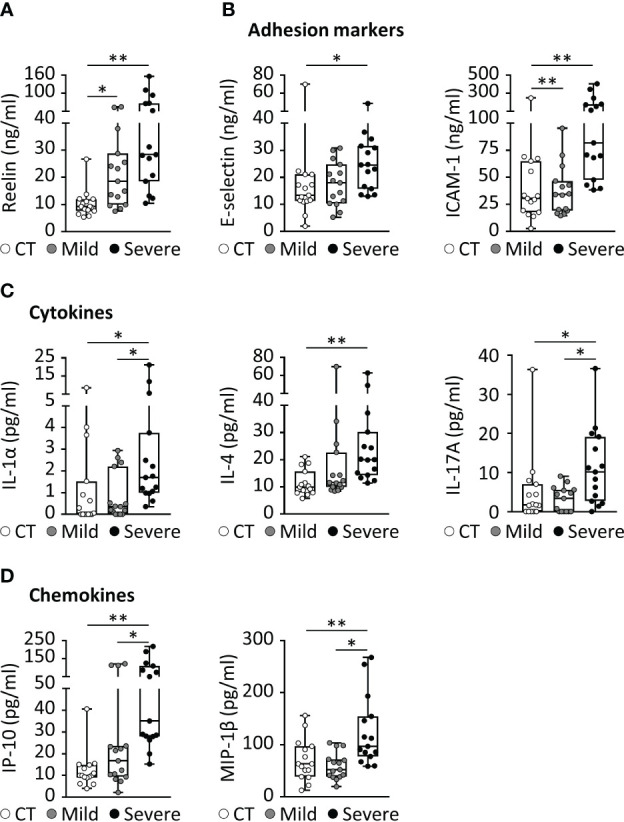
COVID-19 is associated with increased expression of Reelin and inflammatory markers. **(A-D)** Biomarker concentrations were measured in serum from patients with a mild (hospitalized but not ventilated) or severe (hospitalized and ventilated) form of COVID-19. **(A)** Reelin concentration was evaluated by singleplex ELISA. Significant variations from multiplex ELISA are reported for **(B)** adhesion markers, **(C)** cytokines, and **(D)** chemokines. CT, Control healthy patients (age- and gender-matched); *p<0.05, **p<0.01; n=15 per group; detailed cohort is presented in **Table 1**.

Initially discovered for its role in brain development, the glycoprotein Reelin is also recognized as a synaptic homeostatic regulator (31–34). We and others have established that Reelin deficiencies in the brain are correlated to higher cognitive decline in AD patients (35) as well as AD mouse models (35, 36), indicating essential functions in neurons. Surprisingly, we have discovered a completely different and unexpected role for this protein in the systemic circulation, where its expression in the blood increases with inflammation (20). To confirm this observation in COVID-19 patients, we tested Reelin concentration in serum from healthy subjects, COVID-19 patients with a mild form (hospitalization with no ventilation), and COVID-19 patients with a severe form (hospitalization with ventilation). The average Reelin concentration was increased two-fold in the serum of the mild COVID group and five-fold in the severe COVID group, suggesting that Reelin expression increases with the severity of the infection (**Figure 1A** and **Table 1**).

### Circulating Reelin correlates with inflammatory markers in patients with acute COVID-19

**Figure 2:**
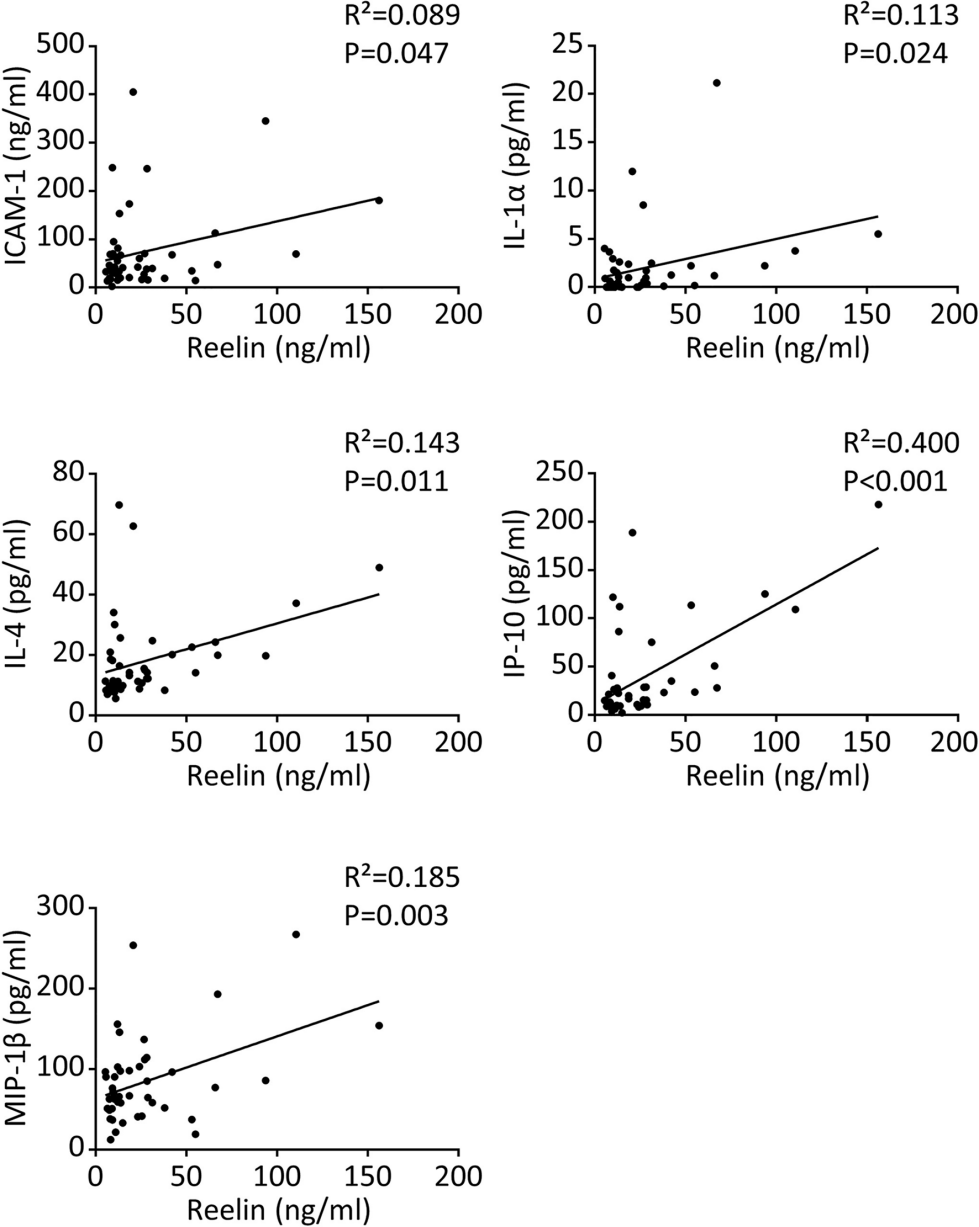
Reelin correlates with inflammatory markers in COVID-19 patients. Significant correlations between Reelin and inflammatory marker concentrations in control and COVID-19 patients. n=15 per group for a total of 45 samples and the detailed cohort is presented in **Table 1**.

Severe COVID-19 reaction is accompanied by the overactivation of inflammatory signals, also known as cytokine storm (5, 7). Therefore, we have measured a large panel of cytokines, chemokines, and adhesion markers to evaluate this dysregulated inflammatory response (**Table 1**, with detailed p values provided in Supplementary **Table 1**). We have also correlated the activation of significant markers with increased Reelin expression. Strikingly, the expression of IL-6 was below the detection limit in control and mild COVID, but increased in the severe group, confirming previous studies (5, 7). TNF-α and IFN-γ were also expected to be elevated during severe COVID (5, 7) but failed to reach a statistically significant increase in our settings. However, a significant elevation of the cytokines IL-1α, IL-4, IL-17A, the chemokines IP-10 (CXCL10), MIP-1β (CCL4), and the adhesion markers E-selectin, ICAM-1 was measured in patients with severe COVID (**Figure 1**). Next, we searched for correlations between Reelin and inflammatory marker concentrations (**Figure 2**). Reelin was significantly associated with IL-1α, IL-4, IP-10, MIP-1β and ICAM-1. Although Reelin is not recognized as an inflammatory marker but rather as a guidance protein or an endothelial activation marker, our results suggest that this circulating protein mirrors the expression of several cytokines, chemokines, and adhesion proteins.

### Long COVID patients do not have residual signs of inflammation

Following SARS-CoV-2-infection, some patients will suffer from PASC that may be due to endothelial injury and dysfunction, cytokine storm, dysregulation of the immune response, or the ability of coronavirus or fragments of it to evade the immune system (8–13). To study the expression of Reelin and inflammatory proteins in these patients, we have used a previously characterized cohort of patients affected by neurological post-acute sequelae of SARS-CoV2 infection (37). Briefly, the cohort presented with symptoms similar to those described in reports of larger cohorts and surveys. Fatigue and cognitive impairment were the most common and debilitating symptoms, with a high prevalence of psychological symptoms and a substantial adverse impact on quality of life. During the acute phase of COVID-19, participants mostly experienced a typical mild disease, except for a relatively high frequency of neurological or psychiatric symptoms.

In PASC patients, all measured parameters were not significantly different from controls, including the markers elevated in severe COVID-19. Of note, only IL-17A and IP-10 showed a trend toward a decline with p=0.06 and p=0.07 respectively, which would be worth investigating further in a larger cohort. This result suggests that there are no longer signs of inflammation or damaged endothelium in long COVID patients.

### Anti-Reelin treatment dampens the disease severity in SARS-CoV-2-infected hACE2 transgenic mice

**Figure 3:**
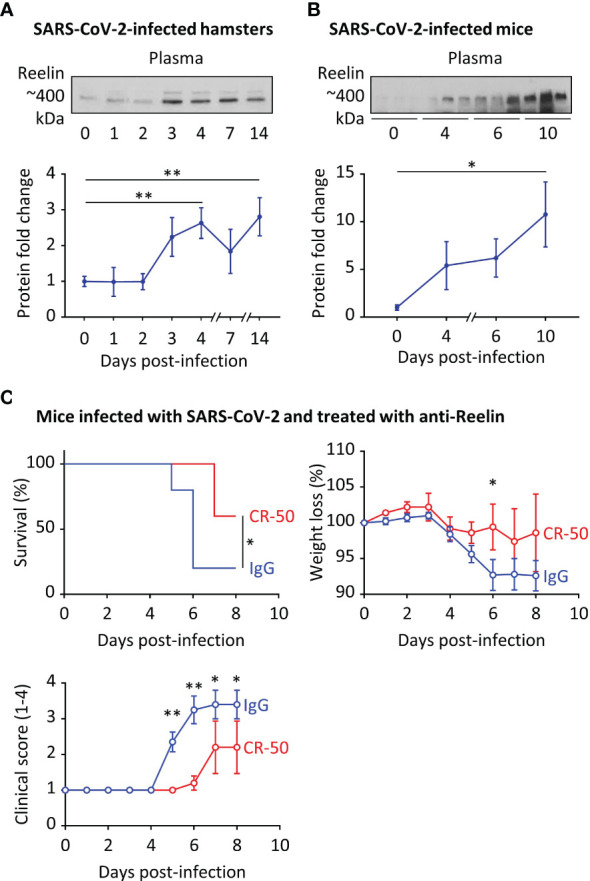
Reelin expression is increased in COVID-19 rodent models and its inhibition dampens the disease severity. **(A)** Plasma Reelin expression was measured over time by western blotting **(A)** in hamsters infected by SARS-CoV-2 (n≥6 per time point) and **(B)** in hACE2 transgenic mice infected by SARS-CoV-2 (n≥3 per time point). **(C)** hACE2 transgenic mice were injected intraperitoneally with an anti-Reelin antibody (CR-50, 100µg, n=5) or a control IgG (100µg, n=10) twice per week, starting 3 days before infection with SARS-CoV-2. Survival, weight loss, and clinical score from 1 (healthy) to 4 (dead) were recorded every day. *p<0.05, **p<0.01.

We observed increased Reelin expression in COVID-19 patients and accordingly, we sought to explore a causal link between this circulating protein and disease severity. For this purpose, we first measured Reelin expression in two COVID-19 rodent models, hamsters and hACE2 transgenic mice that were infected with SARS-CoV-2 (**Figures 3A, B**). In accordance with the human data, plasma Reelin expression started to increase around days 3-4 of infection and remained elevated during the course of the experiment. This increase appears to be associated with the progression of infection in these models. To test whether Reelin depletion might be beneficial, hACE2 transgenic mice were treated with control mouse IgG or anti-Reelin monoclonal mouse antibody (CR-50) at 100µg by intraperitoneal injection twice per week (19–21). This treatment started 3 days before the SARS-CoV-2 infection and clinical parameters were followed for 8 days (**Figure 3C**). CR-50 injections reduced mortality, weight loss, and disease severity as judged by the clinical score. This score is based on a daily clinical assessment of a mouse’s health status, ranging from 1 to 4 as follow: 1 - healthy; 2 - ruffled fur and lethargic behavior; 3 - a score of 2 plus one additional clinical sign such as hunched posture, orbital tightening, increased respiratory rate, and/or > 15% weight loss; 4 - dyspnea and/or cyanosis, reluctance to move when stimulated. These results suggest that CR-50 dampens the severity of SARS-CoV-2 infection and that Reelin has a direct role in the progression of the disease.

## Discussion

In this study, we hypothesized that Reelin promotes endothelial dysfunction and participates in the propagation of hyperinflammation during COVID-19. To address this question, we have shown that this protein is increased in COVID-19 patients and correlates with disease activity, with higher levels in hospitalized individuals with severe respiratory symptoms. In this latter group, we observed a hyperinflammatory state or a cytokine storm, as judged by an increased concentration of serum inflammatory factors, such as cytokines (IL-1α, IL-4, IL-6, IL-10, and IL-17A), chemokines (IP-10, and MIP-1β), and adhesion proteins (E-selectin, and ICAM-1). Reelin level was highly correlated with IL-1α, IL-4, IP-10, MIP-1β, and ICAM-1, suggesting a specific role for Reelin in COVID-19 progression. Furthermore, in a long COVID cohort, Reelin and all the inflammatory mediators above returned to control levels. This shows that the hyperinflammatory state was resolved in these patients. Finally, to establish causality we have tested Reelin inhibition with the anti-Reelin antibody CR-50 in a COVID-19 mouse model. CR-50 prophylactic treatment was able to decrease the mortality and the disease severity in hACE2 transgenic mice infected with SARS-CoV-2. Taken together, these results demonstrate a direct pro-inflammatory function for Reelin in COVID-19 and identify this circulating protein as a potential biomarker and a drug target.

Remarkably, mild COVID and long COVID patients had no significant variation in the expression of inflammatory mediators compared to their controls, and only the severe COVID group showed large changes. In contrast, there was no evidence for an increase in inflammatory factors in the patients with long COVID. This observation is consistent with the description of a cytokine storm, with accumulating studies suggesting that high levels of cytokines are associated with COVID-19 morbidity and mortality in the acute phase of the illness (5–7). When looking specifically at the mild COVID group in our cohort, Reelin stands out as the only marker that is significantly increased (by two-fold). Furthermore, Reelin is increased (by five-fold) in severe COVID, while returning to normal in long COVID cases. This circulating protein appears to be more sensitive than other biomarkers during the low inflammatory state. The singular Reelin expression profile should be further explored in larger cohorts to test its usefulness as a prognostic biomarker.

**Figure 4:**
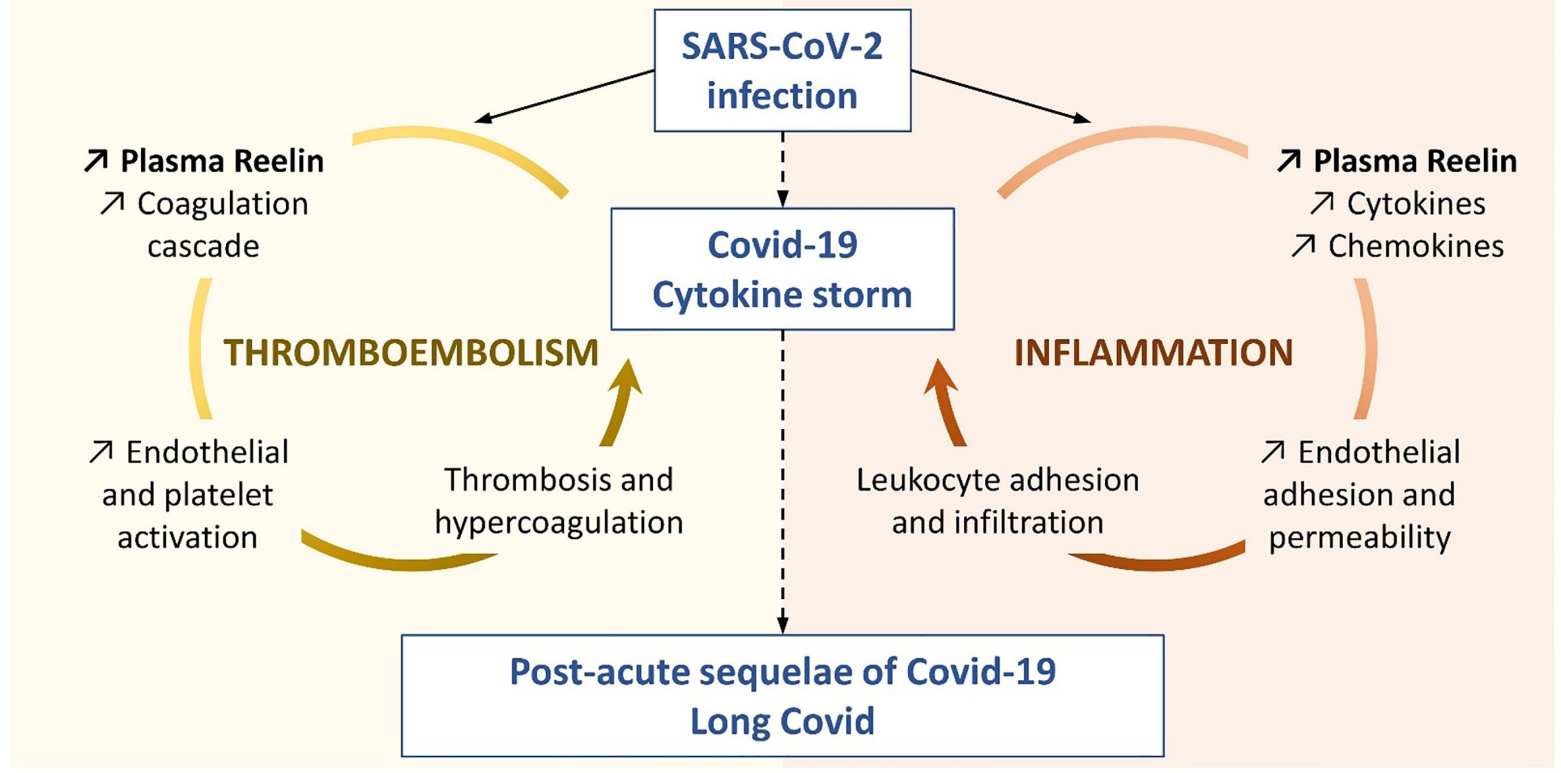
Proposed Reelin mechanism in COVID-19. This representation is based on the literature and the findings presented in this study to illustrate the two functions hypothesized for Reelin in the COVID-19 progression.

For the first time, our study connects circulating Reelin with inflammatory mediators, as demonstrated for IL-1α, IL-4, IP-10 (also known as CXCL10), MIP-1β (also known as CCL4), and possibly IL-6. It suggests that Reelin is altered during inflammation and might regulate or be itself regulated by some inflammatory signals (**Figure 4**). To test this hypothesis, the source of Reelin secretion during inflammation needs to be identified. Candidate sites of expression are hepatic stellate cells (18, 38), kidney, adrenal medulla, vessels, small intestine, submandibular gland, cartilage, and bone (39–41). Besides cytokines and chemokines, Reelin expression also correlated with the adhesion protein ICAM-1, which is consistent with our previous findings. Indeed, several adhesion molecules expressed on the endothelium, including ICAM-1, are regulated by the Reelin/Apoer2/NK-κB axis (18–21). Taken together, these results suggest Reelin as a promoter and biomarker of endothelial dysfunction and potentially of inflammation, which is activated during inflammatory diseases such as atherosclerosis, multiple sclerosis, and now COVID-19. However, it appears that endothelial dysfunction and inflammation no longer persist in long COVID, as the levels of Reelin and inflammatory mediators in these PACS patients are not different from controls. This suggests that this specific pathology is triggered by the acute infection phase and not by chronic systemic inflammation.

Reelin is found in platelets and a role for Reelin in thrombosis has also been established by two independent groups (23–26). Using genetic or antibody-mediated (CR-50) depletion of Reelin in mouse models, these groups demonstrated that Reelin interacts with platelets. This interaction occurs through different receptors and mechanisms such as Glycoprotein VI, α2β3 integrin, phospholipids, thrombin, or FXα. Thereby, Reelin promotes platelet activation leading to thrombin generation and the formation of a fibrin clot. Since Reelin greatly increases with the severity of the infection, its prothrombotic function may participate in or aggravate the thromboembolic complications seen in COVID-19 patients (1–3), and may reflect an extensive platelet activation as seen in the severe forms (42) (**Figure 4**). We have shown previously that Reelin promotes vascular adhesion and permeability to leukocytes, and in this article that Reelin expression in plasma is greatly elevated by ongoing inflammation anywhere in the body. In light of the activity Reelin has on coagulation, this increase in Reelin levels promotes a general prothrombotic state, which, with the initial formation and subsequent expansion of the clot, might lead to further local Reelin release from the aggregated platelets. This in turn would further promote local clotting and thus consolidate the thrombus. Therefore, Reelin functions in a dual capacity, as both a pre-thrombotic clotting facilitator and as a potentiator once an initial clot has formed. Both scenarios are reflected in **Figure 4**.

As discussed above, Reelin promotes vascular adhesion, permeability, and coagulation, which are all clinical hallmarks of severe COVID-19. Moreover, the increase in serum concentration of this protein correlates with the disease severity in infected patients. Therefore, to establish a causal relationship with this pathology, we depleted Reelin using the monoclonal mouse antibody CR-50 in hACE2 transgenic mice infected with SARS-CoV-2. This prophylactic treatment dampens the severity of the infection, as judged by mortality, weight loss, and clinical score. Thus, it demonstrates a direct role for Reelin in the progression to severe COVID-19, potentially through the promotion of endothelial dysfunction and coagulation. In our previous publications on various inflammatory models (atherosclerosis or multiple sclerosis), CR-50 treatment was able to deplete Reelin from the circulation, normalize the expression of endothelial adhesion proteins, decrease the rolling/adhesion/infiltration of leukocytes, and reduce the inflammation (19, 20). Moreover, it is important to note that Reelin is essential for synaptic plasticity (43, 44), migration of neuroblasts (45), as well as dendrite (46) and dendritic spine (47) formation. Therefore, preserving Reelin in the brain is crucial and in our previous studies, we have reported that Reelin expression and function in the brain were not affected by peripheral Reelin depletion. Taken together, these results suggest that the anti-Reelin strategy is relevant in humans and effective in preventing the progression from mild to severe COVID-19, which is characterized by a transition to a hyperinflammatory state (5–7).

This work was designed as a proof-of-concept study to test the relevance of Reelin as a biomarker and biotarget in COVID-19-related pathologies. Thus, it contains some limitations, due to the limited size of human and animal cohorts, as well as the use of animals to model a human disease. For example, a larger cohort might reach a statistically significant variation for IL-17A and IP-10 in the long COVID group, although there was no evidence for overwhelming ongoing inflammation in this group. It would also be more informative in a future study to follow the same patients during the acute phase of the infection and the post-COVID condition. Moreover, the multiplex inflammatory assay selected for this study contains a selection bias and it would be interesting to measure different inflammatory biomarkers commonly used in cardiovascular diseases, such as Galectin-3 (48–56). Finally, the severity of the infection in hACE2 transgenic mice led to a high mortality rate, with the number of animals reaching the endpoint too small for consistent histological and mechanistical analysis. Therefore, future studies should be designed to overcome these limitations and explore in-depth the mechanism by which Reelin contributes to the hyperinflammatory state during COVID-19 infection, where the current literature on Reelin implicates a role in endothelial activation, leukocyte recruitment, and thrombosis.

## Conclusion

In conclusion, this report shows that Reelin expression in plasma is increased during COVID-19 and correlates with disease activity. This large circulating protein is known to regulate endothelial adhesion and permeability to leukocytes, as well as coagulation (**Figure 4**). These two mechanisms are important for COVID-19 progression to a hyperinflammatory state. Furthermore, this study demonstrates that anti-Reelin treatment dampens the severity of the infection in a preclinical model. A panel of cytokines, chemokines, and endothelial dysfunction biomarkers were also examined in mild, severe, and long COVID patients. The results confirm previous findings on the cytokine storm and endothelial dysfunction that occur only in the most severe cases and showed that systemic inflammation is resolved in post-COVID conditions, despite persistent symptoms.

## Data availability statement

The raw data supporting the conclusions of this article will be made available by the authors, without undue reservation.

## Ethics statement

The studies involving human participants were reviewed and approved by UTSW and NIH. The patients/participants provided their written informed consent to participate in this study. The animal study was reviewed and approved by UTMB.

## Author contributions

LC and JHE obtained funding, conceived the hypothesis and project, designed the study, and interpreted the results. LC designed, performed, and analyzed the experiments. AD, JHS, and C-TT designed and performed the rodent models for COVID-19. YM and AN managed and provided the long COVID human cohort. MK helped with the analysis and interpretation of the anti-Reelin strategy. LC wrote and JHE revised the manuscript. All the authors reviewed the manuscript for interpretation and content.
