# Supplementary material for: Circulating Reelin promotes inflammation and modulates disease activity in acute and long COVID-19 cases
Source: Front Immunol. 2023 Jun 27;14:1185748. doi: 10.3389/fimmu.2023.1185748 (PMC10333573; doi:10.3389/fimmu.2023.1185748)
Supplement: Supplementary file 1 [file Table_1.pdf]

Supplementary data

Circulating Reelin promotes inflammation and modulates disease activity in acute and long Covid-19 cases.

Laurent Calvier, Ph.D.[1,2] *; Aleksandra Drelich, PhD.[3]; Jason Hsu[3]; Chien-Te Tseng, PhD.[3]; Yair Mina, M.D. [4,5]; Avindra Nath, M.D.[4]; Maria Z. Kounnas, Ph.D. [6]; Joachim Herz, M.D.[1,2,7,8]

[1] Department of Molecular Genetics, University of Texas (UT) Southwestern Medical Center, Dallas TX, USA.

[2] Center for Translational Neurodegeneration Research, UT Southwestern Medical Center, Dallas TX, USA.

[3] Department of Microbiology & Immunology, UTMB Health, Galveston TX, USA

[4] National Institute of Neurological Disorders and Stroke, National Institutes of Health, Bethesda MD, USA

[5] Sackler Faculty of Medicine, Tel-Aviv University, Tel-Aviv, Israel

[6] Reelin Therapeutics Inc., La Jolla CA, USA.

[7] Department of Neuroscience, UT Southwestern Medical Center, Dallas TX, USA.

[8] Department of Neurology and Neurotherapeutics, UT Southwestern Medical Center, Dallas TX, USA.

*Corresponding author:

Laurent Calvier, Ph.D.
Molecular Genetics
UT Southwestern Medical Center
5323 Harry Hines Blvd.
Dallas, TX 75390-9046
calvier.laurent@gmail.com

Supplementary Table 1. P values associated with Table 1. CT, control; MC, mild Covid; SC, severe Covid.

Target	P value CT vs MC	P value CT vs SC	P value MC vs SC
Reelin	0.0214	<0.0001	
E-selectin		0.0149	
ICAM-1		0.0009	0.0017
IL-1α		0.0107	0.0224
IL-4		0.0006	
IL-17A		0.0353	0.0182
IP-10		<0.0001	0.0189
MIP-1β		0.0269	0.0041
